# Genome-Wide Association Studies and Candidate Genes for Egg Production Traits in Layers from an F_2_ Crossbred Population Produced Using Two Divergently Selected Chicken Breeds, Russian White and Cornish White

**DOI:** 10.3390/genes16050583

**Published:** 2025-05-15

**Authors:** Natalia A. Volkova, Michael N. Romanov, Alan Yu. Dzhagaev, Polina V. Larionova, Ludmila A. Volkova, Alexandra S. Abdelmanova, Anastasia N. Vetokh, Darren K. Griffin, Natalia A. Zinovieva

**Affiliations:** 1 L. K. Ernst Federal Research Center for Animal Husbandry, Dubrovitsy, Podolsk 142132, Moscow Oblast, Russia; natavolkova@inbox.ru (N.A.V.); alan_dz@inbox.ru (A.Y.D.); volpolina@mail.ru (P.V.L.); ludavolkova@inbox.ru (L.A.V.); abdelmanova@vij.ru (A.S.A.); anastezuya@mail.ru (A.N.V.); 2 School of Natural Sciences, University of Kent, Canterbury CT2 7NJ, Kent, UK; d.k.griffin@kent.ac.uk; 3 Animal Genomics and Bioresource Research Unit (AGB Research Unit), Faculty of Science, Kasetsart University, Chatuchak, Bangkok 10900, Thailand; 4 Royal Veterinary College, University of London, London NW1 0TU, UK

**Keywords:** chicken (*Gallus gallus*), genome-wide association study (GWAS), single nucleotide polymorphisms (SNPs), candidate genes, egg performance

## Abstract

**Background/Objectives**: Finding single nucleotide polymorphisms (SNPs) and candidate genes that influence the expression of key traits is essential for genomic selection and helps improve the efficiency of poultry production. Here, we aimed to conduct a genome-wide association study (GWAS) for egg production traits in an F_2_ resource population of chickens (*Gallus gallus*). **Methods**: The examined F_2_ population was produced by crossing two divergently selected breeds with contrasting phenotypes for egg performance traits, namely Russian White (of higher egg production) and Cornish White (of lower egg production). Sampled birds (*n* = 142) were genotyped using the Illumina Chicken 60K SNP iSelect BeadChip. **Results**: In the course of the GWAS analysis, we were able to clarify significant associations with phenotypic traits of interest and economic value by using 47,432 SNPs after the genotype dataset was filtered. At the threshold *p* < 1.06 × 10^−6^, we found 23 prioritized candidate genes (PCGs) associated with egg weight at the age of 42–52 weeks (*FGF14*, *GCK*), duration of egg laying (*CNTN4*), egg laying cycle (*SAMD12*) and egg laying interval (*PHF5A*, *AKR1B1*, *CALD1*, *ATP7B*, *PIK3R4*, *PTK2*, *PRKCE*, *FAT1*, *PCM1*, *CC2D2A*, *BMS1*, *SEMA6D*, *CDH13*, *SLIT3*, *ATP10B*, *ISCU*, *LRRC75A*, *LETM2*, *ANKRD24*). Moreover, two SNPs were co-localized within the *FGF14* gene. **Conclusions**: Based on our GWAS findings, the revealed SNPs and candidate genes can be used as genetic markers for egg weight and other performance characteristics in chickens to attain genetic enhancement in production and for further genomic selection.

## 1. Introduction

The search for, and identification of, gene variants that underlie the manifestation of selection-significant traits in livestock [1,2] is one of the prerequisites and main goals of genomic selection that aims to improve animal production efficiency [3,4,5]. In egg poultry farming, much attention is paid to the phenotypic and genetic indicators characterizing the productive potential of laying hens [6,7]. Finding a set of characteristics that indicate increased egg yield through targeted selection is crucial [8,9], and some of the main criteria for selecting poultry breeds, lines and crosses for industrial egg production are such traits as egg number (EN) and egg weight (EW) [10,11,12]. The history of the creation and improvement of layer breeds is directly related to selection for the expression of these traits [13,14]. EN produced over a certain period of time per laying hen is an important indicator relevant to the egg industry in terms of profitability and economic efficiency [15]. EW is a primary trait in relation to commercial quality of eggs [16], which determines the sale value of this poultry produce. In addition, larger eggs tend to have higher consumer demand [17].

EN laid by a hen during a certain laying period is largely determined by such characteristics as age at first egg (AFE), duration of egg laying (DEL), and egg laying cycle (ELC) [18,19]. Since the initiation of sexual maturity in females coincides with the start of egg laying, AFE is regarded as an indicator that enables prediction of both the bird’s reproductive capacity and egg performance [20,21,22]. Selection of poultry for this trait when developing industrial egg lines and crosses is of particular significance, since a homogeneity of an industrial population of laying hens in terms of the onset of egg laying makes it possible to synchronize the duration of productive use of the layers and enhance the overall egg productivity of the flock [23]. Length of laying cycles and egg laying interval (ELI) directly affect the number of eggs produced by hens during their productive period. Birds with higher egg production are usually characterized by longer laying cycles and shorter periods without laying [19].

The productive potential of layers and its implications for poultry production conditions depend on many factors such as genotype [24,25,26,27,28], feeding [29,30,31,32,33] and maintenance conditions [34,35,36,37], with the latter including lighting and indoor microclimate, among others [38,39]. Some poultry species have seasonal egg laying, e.g., turkeys [40] and geese [41,42].

**Figure 1:**
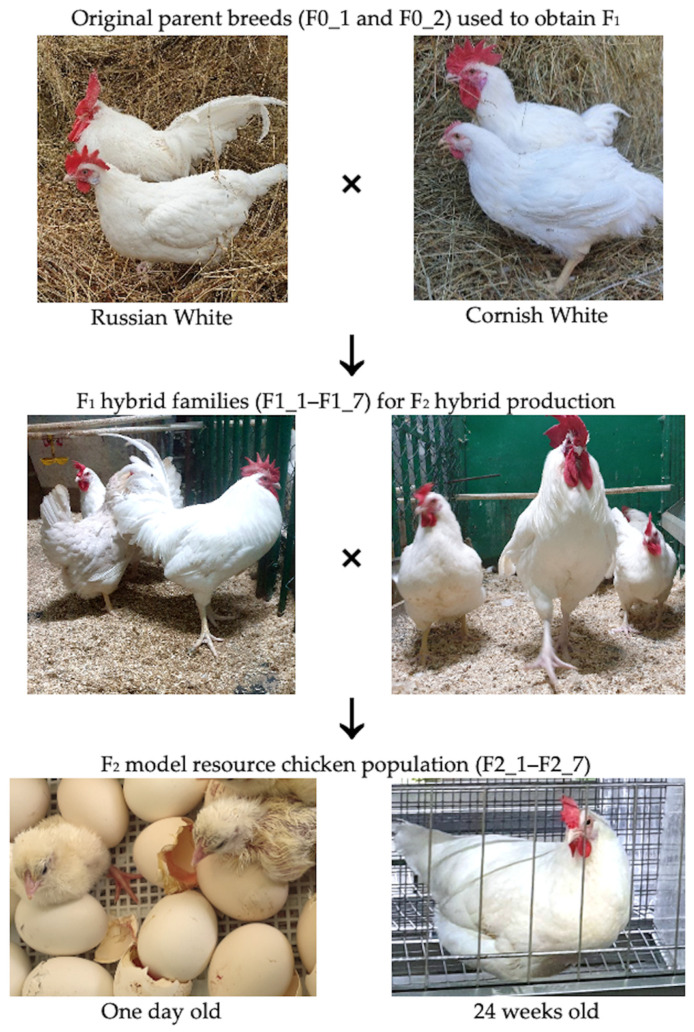
Initial parent breeds and F_1_ hybrids used to obtain the F_2_ model resource chicken population.

The genetic basis of egg performance traits in poultry females has been described in many studies [43,44,45,46,47]. With the advent of high-density single nucleotide polymorphism (SNP) arrays [48], genome-wide association studies (GWAS) have begun to play an essential role in pinpointing previously undetected genetic associations of SNPs and candidate genes with phenotypic traits in chickens [43,49,50,51,52,53], geese [54], quails [55] and ducks [56,57]. Nowadays, GWAS is a molecular tool of choice to investigate genetic blueprints and determinants for various chicken traits of interest, e.g., feed efficiency and growth traits [58], feed conversion ratio [59], muscle fiber and fat traits [60,61], body size [62], semen volume [63], beak deformity [64], response to Newcastle disease [65], and quantitative trait loci (QTL) for ascites syndrome [66], among many others. In our earlier studies using the Russian White (RW) layer breed and the Cornish White (CW) meat breed (Figure 1), we explored putative genes and selective sweeps under strong selection pressure for egg production [67] and identified SNPs and candidate genes associated with AFE in F_2_ hens from a resource population generated by crossing these two breeds [23].

The objective of the present investigation was to extend the above previous works by focusing on SNP discovery and candidate gene identification associated with egg production traits in hens. These traits included AFE, EN, DEL, ELC, ELI, and EW at 18–28 (EW1), 29–41 (EW2) and 42–52 (EW3) weeks of age. To achieve this aim, a GWAS for egg production traits in F_2_ resource chickens from a cross between the RW (of higher egg production) and CW (of lower egg production) breeds (Figure 1) was conducted using whole-genome genotyping (WGG) data.

## 2. Materials and Methods

### 2.1. Experimental Birds

The original breed chickens were reared at the L. K. Ernst Federal Research Centre for Animal Husbandry (LKEFRCAH), hatched from eggs purchased from the Russian Research Institute of Farm Animal Genetics and Breeding (Pushkin, Russia). The F_2_ resource population’s chickens were obtained and raised at the LKEFRCAH (Figure 1), and their DNA was sampled.

To produce the F_2_ chicken resource population, two breeds divergently selected and with contrasting egg production indicators were used, i.e., RW [48,50,68,69] and CW [67,70,71]. At the first stage, based on WGG data and in order to exclude close relationships, individuals of the original parental breeds were divided into two families (F0_1 and F0_2; Figure 1), each consisting of five CW females and one RW male. Each family produced F_1_ hybrids (*n* = 36) through interbreed crosses, which were then selected for further study. These F_1_ interbreed hybrids were utilized to produce F_2_ individuals. For this purpose, seven families (F1_1–F1_7; Figure 1) were formed, each of which included one F_1_ male and three F_1_ females that were not close relatives. The resultant F_2_ offspring (*n* = 142 females; groups F2_1–F2_7; Figure 1) served as a model resource population for additional molecular genetic research aimed at identifying SNPs linked to egg yield indicators of laying hens.

The temperature of the brooders was gradually lowered from 34 °C (in the first few hours after hatching) to 23 °C until the chicks were three weeks old, at which point they were moved to floor maintenance. F_2_ progenies were housed according to their age, which included regular lighting, enough ventilation (to prevent moisture, drafts, and gas pollution), and permanent availability to fresh water and commercial complete compound feed as described elsewhere [72,73]. At the age of 15 weeks, the hens were moved to individual cages to record egg productivity indicators.

### 2.2. Performance Data

The 142 F_2_ females of the resource population were phenotyped for egg production parameters: AFE, EN, DEL, ELC, ELI, plus average EW1, EW2 and EW3. The egg production parameters of each female were recorded individually in the period from the beginning of egg laying to the age of 52 weeks. EN was calculated in the age period from 18 to 52 weeks. ELC was estimated as the period (i.e., number of days) of continuous egg laying, and ELI as the period of absence of egg laying between two ELCs. Eggs were weighed on a laboratory scale. The average EW values were calculated in the first (EW1), second (EW2) and third (EW3) periods of egg laying. The same egg performance indicators (except ELC and ELI) were recorded for chickens of the original parental breeds, i.e., RW (*n* = 20) and CW (*n* = 15).

### 2.3. Sampling and DNA Isolation

Feather pulp was utilized for DNA extraction that was performed using the Syntol animal tissue DNA extraction kit (Syntol, Moscow, Russia). The concentration of DNA solutions was measured using a Qubit 3.0 Fluorimeter (Thermo Fisher Scientific, Wilmington, DE, USA). The NanoDrop-2000 device (Thermo Fisher Scientific) was used to estimate the OD260/280 ratio in order to verify the isolated DNA’s purity.

### 2.4. Genotyping and Quality Control of SNPs

WGG of hens was conducted using the Illumina Chicken iSelect BeadChip DNA array containing ~60K SNPs. In the R-4.0 software environment [74], the PLINK 1.9 software package [75,76] was used to carry out quality control and filtering of WGG data for every sample and every SNP. The following program filters were applied: --mind 0.10, --geno 0.10, --maf 0.01, --hwe 10^−6^. Following SNP pruning, 47,432 SNPs were used in the subsequent analysis.

### 2.5. Principal Component Analysis

Using PLINK, principal component analysis (PCA; [77]) was carried out based on the variance-standardized relationship matrix, and the R program ggplot2 was employed to plot the findings [78,79]. In the R-4.0 software environment [74,80], the data files were created.

### 2.6. Genome-Wide Association Studies

To reveal SNP associations with egg production parameters in F_2_ resource population hens, regression analysis in PLINK 1.9 was executed. The significance of SNP influence and chicken genome regions were identified and evaluated using the Bonferroni null hypothesis test with a *p*-value cutoff (*p* < 1.06 × 10^−6^). The qqman software (version 0.1.9) [81] in the R programming language [82] was used to visualize the data.

The Genome Data Viewer in the NCBI chicken databases [83] and the chicken (*G. gallus*; GGA) reference genome assembly GRCg6a [84] were used to search for putative genes located in the vicinity of the detected SNPs, including 0.2–Mb flanks on both sides. To obtain comprehensive information about SNPs found within or close to the identified candidate genes, the web-based Ensembl Genes release 106 database and the Ensembl BioMart data mining program [85] were used. The Ensembl BioMart data mining tool and Database for Annotation, Visualization, and Integrated Discovery (DAVID Knowledgebase; version DAVID 2021 (December 2021; v2023q4, quarterly updated)) [86,87] were utilized to conduct functional annotation and gene ontology (GO) term enrichment analysis for major candidate genes. The search for associations of identified SNPs and potential genes with selectively significant traits in chickens identified in other studies was carried out in the Animal QTLdb (https://www.animalgenome.org/cgi-bin/QTLdb/index (accessed on 21 April 2025)) and Chicken QTLdb (https://www.animalgenome.org/cgi-bin/QTLdb/GG/index (accessed on 21 April 2025)) databases. All required database data were extracted to build GRCg6a (https://www.animalgenome.org/QTLdb/doc/genome_versions#chicken (accessed on 21 April 2025)) in .bed format, .gff and .sam formats.

## 3. Results

### 3.1. Phenotypic Data and Population Stratification

Table 1 presents descriptive statistics characterizing the distribution of values established for the egg productivity traits studied in F_2_ resource population hens. A higher variability of values was established for a number of traits, e.g., ELC and ELI had coefficients of variation up to 33.4 and 41.4%, respectively.

It should be noted that the obtained F_2_ resource population hens had a tendency for higher variability in the phenotypic expression of the studied egg productivity traits compared to the original parent breeds (Table 1).

**Figure 2:**
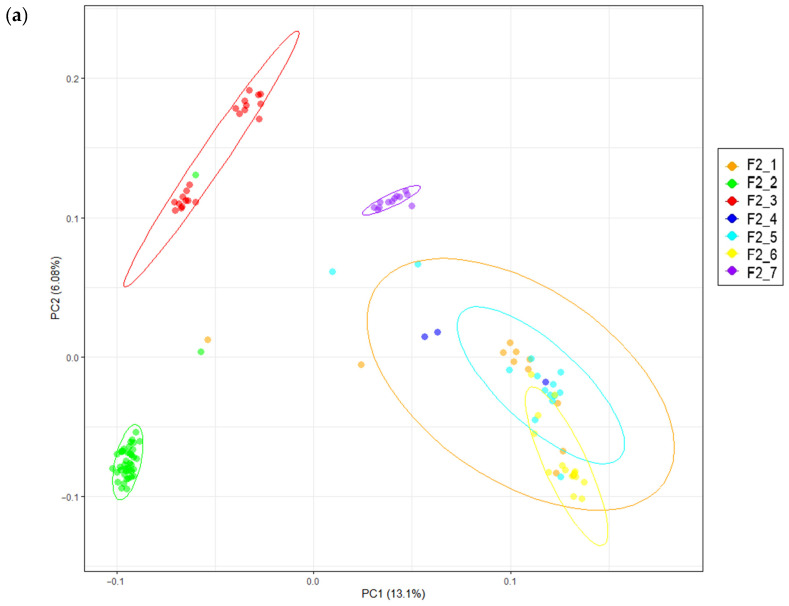

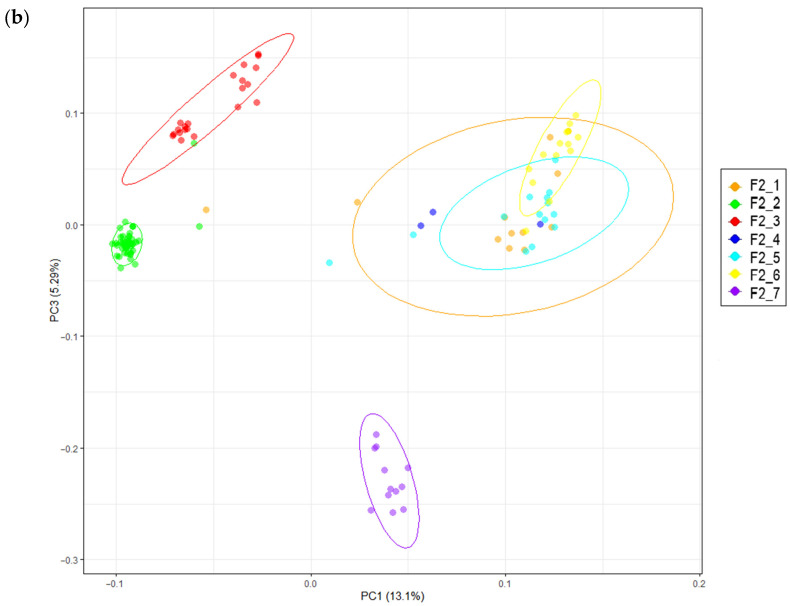
Principal component analysis (PCA) for the F_2_ resource chicken population. (**a**) PCA performed in the projection of the first (PC1) and second (PC2) components. *X*-axis, PC1; *Y*-axis, PC2. (**b**) PCA performed in the projection of the PC1 and third (PC3) components. *X*-axis, PC1; *Y*-axis, PC3. Different colors are used to represent members of certain groups.

The distribution of the examined F_2_ resource population was displayed by PCA across several clusters. In the projections of first component (PC1)–second component (PC2) and PC1–third component (PC3), differentiation of the studied population into four groups was noted, as follows: the first grouping included F2_1, F2_4, F2_5 and F2_6 progenies, the second grouping included F2_2 individuals, the third grouping included F2_3 chickens, and the fourth grouping included F2_7 offspring. These data are shown graphically in Figure 2a,b.

Consequently, we conducted a GWAS trial employing the first three PCs (i.e., PC1, PC2 and PC3) as covariates in light of the observed population stratification, or its disclosed structure.

### 3.2. Genome-Wide Association Analysis Output

**Figure 3:**
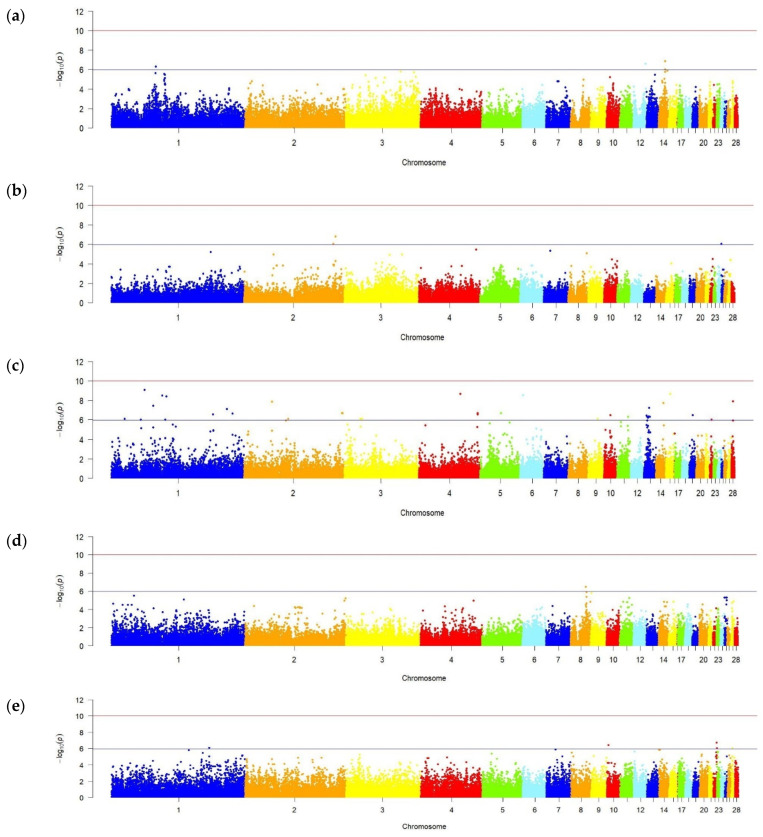
Manhattan plots resulting from GWAS for the studied egg production traits in the F_2_ resource chicken population. (**a**) Duration of egg laying from age at first egg to 52 weeks of age; (**b**) egg laying cycle; (**c**) egg laying interval; (**d**) mean egg weight at 18–28 weeks of age; and (**e**) mean egg weight at 42–52 weeks of age. Manhattan plots: distribution of single nucleotide changes across 28 chicken chromosomes (autosomes) at the significance level (−log_10_(*p*)) based on the traits’ predicted probability values. The only purpose of color-coding dots is to show chromosomal segregation.

The obtained phenotypic data on egg production parameters in F_2_ resource population hens (Table 1) were used for the subsequent GWAS. Figure 3 presents the respective GWAS results.

The completed analysis discovered six SNPs associated with EN in hens of the resource population and EW in different periods of their egg laying, and 45 SNPs associated with the studied DEL and ELI parameters at the level of established significance value *p* < 1.06 × 10^−6^ (Supplementary Table S1). These SNPs were identified on 19 of 28 chromosomes explored. The maximum number of significant SNPs was localized on GGA1 (14 SNPs), and the minimum (1 SNP) on GGA5, GGA6, GGA8, GGA9, GGA11, GGA12, GGA15, GGA19, GGA25, GGA27 and GGA28. No significant SNPs were identified on chromosomes GGA7, GGA16–GGA18, GGA20, GGA21, GGA23, GGA24 and GGA26 for any of the studied parameters.

Information about the distribution and quantity of significant SNPs found on chromosomes, taking into account each specifically studied egg productivity indicator in F_2_ resource population hens, is shown in Table 2.

The number of significant SNPs associated with the studied egg laying parameters (DEL, ELI, ELC and ELI) varied from 3 to 38. A significant proportion of these SNPs were localized on chromosomes GGA1, GGA4 and GGA13 (5–12 SNPs).

GWAS of EW parameters in the studied F_2_ population hens revealed one and five SNPs, respectively, associated with this trait in the first (18–28 weeks) and third (42–52 weeks) periods of egg laying. These SNPs were established on five chromosomes (GGA8, GGA1, GGA10, GGA22, and GGA27).

For three indicators (EN, AFE and EW2), no significant SNPs were identified in this study at the established significance threshold.

### 3.3. Candidate Genes

Candidate genes linked to the studied egg productivity parameters in F_2_ resource population hens were annotated using the significant SNPs that were found. In the regions of identified SNPs (i.e., SNP position ± 0.2 Mb), a total of 219 genes described in the NCBI databases [83,84] were detected (Supplementary Table S1), including 23 prioritized candidate genes (PCGs) within which the identified SNPs were localized. The latter genes were found on 13 chromosomes (GGA1–GGA4, GGA6, GGA10–GGA13, GGA15, GGA19, GGA22 and GGA28). In the case of one PCG, for fibroblast growth factor 14 (*FGF14*), two SNPs associated with EW3 were identified. Significant SNPs (*p* < 1.06 × 10^−6^) associated with egg performance of F_2_ resource population hens and the appropriate candidate genes are shown in Table 3.

The annotated genes were categorized into four functional clusters according to the GO term enrichment score. Three clusters, however, were deemed insignificant since their enrichment scores were less than one. The remaining one cluster (with enrichment scores > 1.15) included genes related to protein phosphorylation and ATP binding.

Supplementary Table S2 lists every gene that was annotated along with its function. Comparative analysis of the results obtained in this study with information available in the Animal QTLdb database (https://www.animalgenome.org/cgi-bin/QTLdb/index (accessed on 21 April 2025)) confirmed the significant association of four identified candidate genes with selectively important traits in chickens, including five genes with egg production indicators (Supplementary Table S3).

## 4. Discussion

Elucidating the genetic basis for efficient poultry breeding involves the search for, and identification of, valuable genotypes focusing on the use of genomic technologies [88,89]. This has become possible thanks to the initial generation of the complete chicken genome sequence [90,91] that contributed to advances in the genomics of other bird species [92,93,94,95,96,97,98]. For the successful implementation of genomic technologies in poultry farming practice, it is essential to explore the molecular genetic mechanisms underlying the phenotypic variability of economically important traits that are crucial for improving the efficiency of agriculture and increasing the production of competitive products [99,100]. Our work presented here yielded the associations of SNPs and candidate genes with the main egg productivity traits in F_2_ chickens of the resource population produced through interbreeding of the divergently selected breeds, RW and CW, that contrast in egg performance.

### 4.1. Egg Number and Egg Weight

EN and EW are the main egg performance indicators for which egg breeds are selected [101]. Intensive selection of chickens for these traits contributed to the creation of highly productive egg crosses, from which more than 300 eggs are expected per year per layer [9]. At the same time, long-term selection of chickens for high egg production contributed to a decline in genetic variability for this trait [102]. To date, many studies have been conducted aimed at finding candidate genes associated with EN [103,104,105,106,107] and EW [108,109,110,111].

In our study, two PCGs were identified that were associated with EW3, including the *FGF14* gene [112] and the glucokinase (*GCK*) gene [113]. To the best of our knowledge, previous investigations have not revealed any direct connection between these genes and EW in chickens. At the same time, in research by Li et al. [114], the influence of *FGF14* on the eggshell qualitative characteristics in hens in the late period of egg laying was demonstrated. These authors described the expression profiles of mRNAs and long non-coding RNAs (lncRNAs) in the eggshell glands of young and older laying chickens, comparing each in order to find potential genes linked to aging in the laying hen’s uterus. The eggshell’s thickness correlates with its strength [115] and also determines the weight indicators of both the shell and the whole egg. Taking this into account, the results of that study [115] can be considered as an indirect confirmation of the *FGF14* gene association with EW established in our investigation.

The *GCK* gene enables glucokinase activity by its involvement in the glucose 6-phosphate metabolic process and in the glycolytic process. A number of studies have shown the involvement of *GCK* in the glycolytic process in poultry, in particular, in chickens [116,117] and ducks [118]. Its relationships with feed consumption [116,117], embryo development, muscle development and egg production in chickens [119] have been established. Christensen et al. [120] established a positive correlation between high glucose concentration, on the one hand, and survival, weight and rapid growth of embryos and hatched turkey chicks, on the other. The weight of the hatching egg is positively correlated with the development and weight of embryos [121] and hatched chicks [122,123]. Based on this, it can be assumed that *GCK* may indirectly affect EW.

### 4.2. Age at First Egg and Duration of Egg Laying

AFE, or the start of egg laying in poultry, relates to the sexual maturation process of growing birds and the commencement of sexual maturity, which are both controlled by the hypothalamic–pituitary–gonadal system [20,124]. Many studies have shown the influence of the hypothalamic–pituitary–gonadal axis on hormones and genes related to egg production indicators, including AFE, in various poultry species [20,21,22].

Previously, we demonstrated the association of the genes for axin interactor, dorsalization associated (*AIDA*), Na^+^/K^+^ transporting ATPase interacting 2 (*NKAIN2*), lin-9 DREAM MuvB core complex component (*LIN9*) and mitogen-activated protein kinase kinase kinase kinase 3 (*MAP4K3*) with AFE [23]. In the present work, we did not identify genes associated with AFE at the level of the significance threshold established in this study. One respective PCG, contactin 4 (*CNTN4*), was identified on GGA12 that was associated with DEL. This productive trait is directly related to AFE [20,124]. Liu et al. [125] established a significant association between *CNTN4* and feed conversion ratio in an F_2_ resource population of laying ducks during the egg laying period. In the same experiment, correlations were found between the feed conversion ratio and egg production traits. This may partly confirm our results on the association of *CNTN4* with DEL, since egg production determines the duration of the productive use of laying hens.

### 4.3. Egg Laying Cycle and Egg Laying Interval

The duration of ELCs and ELIs are important selection indicators that determine the egg productivity and the productive use duration of poultry, including chickens [126,127,128]. The implementation of the productive potential of laying hens in terms of these indicators depends on a number of factors, including the development and functional characteristics of the reproductive organs associated with egg laying [102,129], as well as feed intake [126,130,131,132,133,134] and housing conditions [39,135].

Relative to our experimental data, a comparative analysis of open information sources, e.g., the Chicken QTLdb database (https://www.animalgenome.org/cgi-bin/QTLdb/GG/index (accessed on 21 April 2025)), demonstrated that, for eight PCGs identified in our research, other studies have also shown close relationships with selection-significant traits in chickens. These PCGs included ATPase copper transporting beta (*ATP7B*), aldo-keto reductase family 1, member B10 (aldose reductase) (*AKR1B1*), caldesmon 1 (*CALD1*), slit guidance ligand 3 (*SLIT3*), coiled-coil and C2 domain containing 2A (*CC2D2A*), BMS1, ribosome biogenesis factor (*BMS1*), semaphorin 6D (*SEMA6D*), and ATPase phospholipid transporting 10B (putative) (*ATP10B*) that were related to egg laying performance in F_2_ resource population hens. In particular, two genes are known to be associated with the development of reproductive organs and egg laying in chickens, i.e., *CALD1* with the morphogenesis of the Müllerian ducts, which develop into the reproductive tract of female vertebrates [136], and *SLIT3* with ovarian follicle growth [137]. Also, other studies reported associations of these genes with eggshell quality characteristics such as eggshell strength [138,139,140], and eggshell effective layer thickness (https://www.animalgenome.org/cgi-bin/QTLdb/GG/qdetails?QTL_ID=159369 (accessed on 21 April 2025); [141]).

Curiously, the work presented here has parallels beyond birds into mammalian egg production. In sheep oocytes, a significant upregulation of the *AKR1B1* gene was observed in the warm season [142], which may be suggestive of its role in the reproductive cycle of homeothermic species. In the works of Jehl et al. [143] and Yuan et al. [144], a search was performed for candidate genes associated with the efficiency of daily feed intake in laying hens. A definite association was established for *AKR1B1* [143] and *ATP7B* [144] with this indicator. Also, for the genes associated in our study with ELI, other works reported their relationship with poultry growth and meat productivity. In particular, *AKR1B1* was shown to be associated with growth and abdominal fat deposition [145], and with chest width in 7-week-old chickens (https://www.animalgenome.org/cgi-bin/QTLdb/GG/qdetails?QTL_ID=257052 (accessed on 21 April 2025); [146]). Furthermore, associations were suggested for *BMS1* [147], *ATP10B* [51] and *ATP7B* (https://www.animalgenome.org/cgi-bin/QTLdb/GG/qdetails?QTL_ID=261257 (accessed on 21 April 2025); [148]) with chicken growth rates; *SEMA6D* with growth and body weight in laying ducks aged 18 weeks [149]; and *CC2D2A* with shank weight, tibia weight and femur weight in ducks during the laying period [150].

With all the above in mind, the available results of other studies largely agree with our data on the direct effect of the *CALD1*, *SLIT3* and *FGF14* genes on the egg productivity indices of hens. For other genes identified in our work, a number of studies have also shown their relationship with growth and meat productivity indices (*ATP7B*, *CC2D2A*, *AKR1B1*, *SLIT3*, *BMS1* and *ATP10B*), feed consumption of laying hens during the egg laying period (*CNTN4*, *ATP7B* and *AKR1B1*) in chickens, growth indices (*SEMA6D*), and the state of the musculoskeletal system (*CC2D2A*) in laying ducks. Based on the hypothesis that genes interrelating in similar biological networks may collectively affect the egg production phenotype [151], we also examined all genes that overlapped the significant SNP regions that we identified in our GWAS for functional enrichment (Supplementary Table S2). According to GO analysis, the best candidates were enriched for protein phosphorylation and ATP binding, and calcium ion binding, which have broad biological/metabolic functions and roles [85,86,87]. Further studies using GWAS, whole-genome sequencing and other approaches (e.g., [104,152,153,154,155]) are required to confirm the association of these potential genes with egg production in layers and elucidate endocrine and genetic factors affecting egg laying performance [106].

## 5. Conclusions

In this investigation, we performed a GWAS for traits associated with egg production in an F_2_ resource population using the Illumina Chicken 60K SNP iSelect BeadChip. The conducted studies revealed 51 SNPs and 23 PCGs showing a significant association with EW1 (one SNP) and EW3 (five SNPs; *FGF14* and *GCK* genes), as well as with DEL (four SNPs; *CNTN4* gene), ELC (three SNPs; *SAMD12* gene), and ELI (38 SNPs; *PHF5A*, *AKR1B1*, *CALD1*, *ATP7B*, *PIK3R4*, *PTK2*, *PRKCE*, *FAT1*, *PCM1*, *CC2D2A*, *BMS1*, *SEMA6D*, *CDH13*, *SLIT3*, *ATP10B*, *ISCU*, *LRRC75A*, *LETM2*, and *ANKRD24* genes) in the studied birds. The maximum number of identified SNPs and candidate genes was detected on chromosome GGA1 (14 SNPs), while the minimum number was observed on chromosomes GGA5, GGA6, GGA8, GGA9, GGA11, GGA12, GGA15, GGA19, GGA25, GGA27 and GGA28 (one SNP each). In one of the 23 identified PCGs, *FGF14*, two SNPs were localized associated with EW3. These findings are crucial for comprehending the molecular genetic underpinnings of development and implementation of productive potential in hens. Although they need more research, the discovered SNPs and PCGs can be utilized as genetic markers in breeding initiatives meant to boost and enhance egg yield.

## Figures and Tables

**Table 1:** Descriptive statistics for egg production indicators ^1^ in F_2_ resource population hens as compared to the original parent breeds.

Trait	F_2_ Population (*n* = 142)		Russian White (*n* = 20)		Cornish White (*n* = 15)	
	Mean ± SD	CV, %	Mean ± SD	CV, %	Mean ± SD	CV, %
Age at first egg, days	131.6 ± 18.4	13.9	144.5 ± 6.3	4.4	180.6 ± 17.8	9.9
Duration of egg laying, days	232.4 ± 18.4	7.9	220.5 ± 6.3	2.9	184.4 ± 17.8	9.6
Egg number for 238 days	126.2 ± 31.6	25.1	150.3 ± 10.8	7.2	82.0 ± 11.1	13.5
Egg laying cycle, days	2.4 ± 0.8	33.4	N/A	N/A	N/A	N/A
Egg laying interval, days	2.4 ± 1.0	41.4	N/A	N/A	N/A	N/A
Egg weight at age of 18–28 weeks, g	43.0 ± 3.5	8.2	44.1 ± 3.2	7.2	N/A	N/A
Egg weight at age of 29–41 weeks, g	50.8 ± 4.7	9.3	50.6 ± 4.3	8.7	52.2 ± 4.2	8.0
Egg weight at age of 42–52 weeks, g	57.8 ± 3.9	6.7	55.1 ± 2.4	4.3	63.1 ± 4.6	7.4

^1^ SD, standard deviation; CV, coefficient of variation; *n*, number of females; N/A, not available due to unavailability of individual trait recording.

**Table 2:** Chromosomal distribution of significant SNPs (*p* < 1.06 × 10^−6^) associated with egg production indicators in F_2_ resource population hens.

Trait	No. of SNPs	Chromosomes ^1^
Duration of egg laying	4	GGA1, GGA12, GGA14
Egg laying cycle	3	GGA2, GGA25
Egg laying interval	38	GGA1–GGA6, GGA9–GGA11, GGA13–GGA15, GGA19, GGA22, GGA28
Egg weight at age of 18–28 weeks	1	GGA8
Egg weight at age of 42–52 weeks	5	GGA1, GGA10, GGA22, GGA27

^1^ GGA, chicken (*G. gallus*) chromosome.

**Table 3:** Significant SNPs and prioritized candidate genes (in SNP positions) associated with egg production indicators in F_2_ resource population hens.

Trait	GGA ^1^	SNP	Location, bp	Gene	*p*-Value
Egg weight at 42–52 weeks of age	1	Gga_rs13950763	144,541,672	*FGF14*	7.893 × 10^−7^
		Gga_rs13950783	144,581,256	*FGF14*	7.893 × 10^−7^
	22	Gga_rs16733701	5,254,215	*GCK*	8.868 × 10^−7^
Duration of egg laying	12	Gga_rs14048080	18,439,246	*CNTN4*	2.497 × 10^−7^
Egg laying cycle	2	Gga_rs13772998	136,095,111	*SAMD12*	1.565 × 10^−7^
Egg laying interval	1	GGaluGA016975	49,631,028	*PHF5A*	8.431 × 10^−10^
		GGaluGA021889	62,111,416	*AKR1B1*	3.519 × 10^−8^
		Gga_rs14835481	62,385,811	*CALD1*	4.072 × 10^−15^
		Gga_rs13973123	171,655,178	*ATP7B*	7.489 × 10^−8^
	2	Gga_rs13670867	41,560,503	*PIK3R4*	1.337 × 10^−8^
		Gga_rs14258322	145,755,426	*PTK2*	2.003 × 10^−7^
	3	Gga_rs14329753	26,472,162	*PRKCE*	7.511 × 10^−7^
	4	Gga_rs15598417	61,793,533	*FAT1*	2.166 × 10^−9^
		Gga_rs15600128	62,825,026	*PCM1*	4.072 × 10^−15^
		GGaluGA266321	76,726,036	*CC2D2A*	5.887 × 10^−15^
	6	Gga_rs14564900	5,790,608	*BMS1*	2.811 × 10^−9^
	10	GGaluGA068824	10,301,717	*SEMA6D*	3.172 × 10^−7^
	11	GGaluGA078973	16,137,404	*CDH13*	4.874 × 10^−7^
	13	GGaluGA092132	5,413,088	*SLIT3*	3.573 × 10^−7^
		Gga_rs14050895	8,419,842	*ATP10B*	4.550 × 10^−7^
	15	Gga_rs15773720	6,817,919	*ISCU*	2.166 × 10^−9^
	19	GGaluGA126763	5,248,070	*LRRC75A*	3.247 × 10^−7^
	22	Gga_rs14684608	2,639,829	*LETM2*	9.628 × 10^−7^
	28	Gga_rs16210664	2,694,485	*ANKRD24*	1.184 × 10^−7^

^1^ GGA, chicken (*G. gallus*) chromosome.

## Data Availability

The genotyping data presented in this study can be shared with the third parties upon approval with the GWMAS Consortium. Other original contributions presented in the study are included in the article and Supplementary Materials; further inquiries can be directed to the corresponding authors with the permission provided by the chickens’ owners.

## Supplementary Materials

The following supporting information can be downloaded at: https://www.mdpi.com/article/10.3390/genes16050583/s1, Table S1: SNPs and candidate genes associated with egg production parameters in F_2_ resource population hens; Table S2: Gene ontology (GO) term enrichment analysis at the positions of the determined SNPs in F_2_ resource population chickens; Table S3: Comparative analysis of the obtained experimental data relative to the Animal QTLdb database (https://www.animalgenome.org/cgi-bin/QTLdb/index).
